# Supplementary material for: A semiconductor 96-microplate platform for electrical-imaging based high-throughput phenotypic screening
Source: Nat Commun. 2023 Nov 21;14:7576. doi: 10.1038/s41467-023-43333-9 (PMC10663594; doi:10.1038/s41467-023-43333-9)
Supplement: Supplementary file 3 — Description of additional supplementary files [file 41467_2023_43333_MOESM3_ESM.pdf]

Description of additional supplementary files

Supplementary Movie 1: Growth characteristics of eleven cell lines at different densities over 48 hours. Cells were plated at the densities indicated in Supplementary Figure 6. Densities are laid out from low to high, top to bottom in each column for the indicated cell type. Videos were generated with VF 250 Hz in red, VF 16 kHz in green, and the inverse of the LF 16 kHz in blue. Time ranges from 0- 48 hours post seeding.

Supplementary Movie 2: Real-time compound responses in MDCK cells. MDCK cells were plated and treated with the indicated compounds and concentration in triplicate replicates corresponding to traces shown in Supplementary Figure 8. Cells were seeded at time t = 0, compounds were added at time t = 27 hours. Effects measured up to 48 hours post compound addition. Videos were generated with VF 250 Hz in red, VF 16 kHz in green, and the inverse of the LF 16 kHz in blue.

Supplementary Movie 3: Real-time compound responses in A549 cells. A549 cells were plated and treated with the indicated compounds and concentration in triplicate corresponding to traces shown in Supplementary Figure 9. Cells were seeded at time t = 0, compounds were added at time t = 27 hours. Effects measured up to 48 hours post compound addition. Videos were generated with VF 250 Hz in red, VF 16 kHz in green, and the inverse of the LF 16 kHz in blue.

Supplementary Movie 4: Real-time compound responses in MDA-MB231 cells. MDA-MB-231 cells were plated and treated with the indicated compounds and concentration in triplicate corresponding to traces shown in Supplementary Figure 10. Cells were seeded at time t = 0, compounds were added at time t = 26 hours. Effects measured up to 48 hours post compound addition. Videos were generated with VF 250 Hz in red, VF 16 kHz in green, and the inverse of the LF 16 kHz in blue.
