# Supplementary material for: A semiconductor 96-microplate platform for electrical-imaging based high-throughput phenotypic screening
Source: Nat Commun. 2023 Nov 21;14:7576. doi: 10.1038/s41467-023-43333-9 (PMC10663594; doi:10.1038/s41467-023-43333-9)
Supplement: Supplementary file 8 — Reporting Summary [file 41467_2023_43333_MOESM8_ESM.pdf]

nature portfolio

Corresponding author(s): Jeffrey Abbott

Last updated by author(s): Jul 24, 2023

Reporting Summary

Nature Portfolio wishes to improve the reproducibility of the work that we publish. This form provides structure for consistency and transparency in reporting. For further information on Nature Portfolio policies, see our Editorial Policies and the Editorial Policy Checklist.

Statistics

For all statistical analyses, confirm that the following items are present in the figure legend, table legend, main text, or Methods section.

n/a	Confirmed	
☐	☒	The exact sample size (*n*) for each experimental group/condition, given as a discrete number and unit of measurement
☒	☐	A statement on whether measurements were taken from distinct samples or whether the same sample was measured repeatedly
☒	☐	The statistical test(s) used AND whether they are one- or two-sided<br>*Only common tests should be described solely by name; describe more complex techniques in the Methods section.*
☒	☐	A description of all covariates tested
☐	☒	A description of any assumptions or corrections, such as tests of normality and adjustment for multiple comparisons
☐	☒	A full description of the statistical parameters including central tendency (e.g. means) or other basic estimates (e.g. regression coefficient) AND variation (e.g. standard deviation) or associated estimates of uncertainty (e.g. confidence intervals)
☒	☐	For null hypothesis testing, the test statistic (e.g. *F*, *t*, *r*) with confidence intervals, effect sizes, degrees of freedom and *P* value noted<br>*Give P values as exact values whenever suitable.*
☒	☐	For Bayesian analysis, information on the choice of priors and Markov chain Monte Carlo settings
☐	☒	For hierarchical and complex designs, identification of the appropriate level for tests and full reporting of outcomes
☒	☐	Estimates of effect sizes (e.g. Cohen's *d*, Pearson's *r*), indicating how they were calculated

Our web collection on statistics for biologists contains articles on many of the points above.

Software and code

Policy information about availability of computer code

Data collection	Custom code was used to interface with the electronic plate, run the measurements and collect the data. Custom data pipeline was used to output individual and aggregate pixel values from the raw electronic data.
Data analysis	Data outputs were saved as csvs and analyzed using custom Python code.All plots shown in figures were generated by matplotlib, seaborn and plotly. For the compound profiling analysis (Figure 6), we performed PCA, Agglomerative clustering and LDA, model validation using the sklearn package. Cell-line functional indices (Fig. 3c,d) and bio-basis (Fig 6e) calculations are described in the methods.Fiji was used to compile immunofluorescence images for publication.

For manuscripts utilizing custom algorithms or software that are central to the research but not yet described in published literature, software must be made available to editors and reviewers. We strongly encourage code deposition in a community repository (e.g. GitHub). See the Nature Portfolio guidelines for submitting code & software for further information.

Data

Policy information about availability of data

All manuscripts must include a data availability statement. This statement should provide the following information, where applicable:

- Accession codes, unique identifiers, or web links for publicly available datasets
- A description of any restrictions on data availability
- For clinical datasets or third party data, please ensure that the statement adheres to our policy

The impedance data generated in this study is included in the Source Data file. Source data are provided with this paper.

Research involving human participants, their data, or biological material

Policy information about studies with human participants or human data. See also policy information about sex, gender (identity/presentation), and sexual orientation and race, ethnicity and racism.

Reporting on sex and gender	NA
Reporting on race, ethnicity, or other socially relevant groupings	NA
Population characteristics	NA
Recruitment	NA
Ethics oversight	NA

Note that full information on the approval of the study protocol must also be provided in the manuscript.

Field-specific reporting

Please select the one below that is the best fit for your research. If you are not sure, read the appropriate sections before making your selection.

- [x] Life sciences
- [ ] Behavioural & social sciences
- [ ] Ecological, evolutionary & environmental sciences

For a reference copy of the document with all sections, see nature.com/documents/nr-reporting-summary-flat.pdf

Life sciences study design

All studies must disclose on these points even when the disclosure is negative.

Sample size	No sample size calculation was performed. All experiments were done in technical and biological replicates. Variation across technical replicates was low (further illustrated in Supp Fig 14.), and three technical replicates was considered sufficient. To demonstrate biological effects, experiment was repeated 3 times. Results presented showed consistent response across repllcates. High-throughput screen was performed as a single replicate.
Data exclusions	Data from wells that were electronically faulty was excluded from the analysis. Parameters for exclusion were predefined. Electronically faulty wells resulted in no signal/ saturating signal.
Replication	All experiments with the exception of the high-throughput screen were performed in triplicates. For certain key experiments, biological replicates were performed to verify results. All responses described in the manuscripts were reproduceable. To highlight reproducibility across technical and biological replicates, we performed an analysis of the positive controls as described in Figure 6.
Randomization	Our research did not involve organisms/participants or samples in which randomization would be relevant. In the described high-throughput screen, compound allocation by plate was random. Randomization was included in the library design.
Blinding	No blinding was performed in this study. Readouts from the platform are automated, not subject to bias from the researcher. Thus blinding was not considered necessary.

Reporting for specific materials, systems and methods

We require information from authors about some types of materials, experimental systems and methods used in many studies. Here, indicate whether each material, system or method listed is relevant to your study. If you are not sure if a list item applies to your research, read the appropriate section before selecting a response.

Materials & experimental systems

n/a	Involved in the study	
☐	☒	Antibodies
☐	☒	Eukaryotic cell lines
☒	☐	Palaeontology and archaeology
☒	☐	Animals and other organisms
☒	☐	Clinical data
☒	☐	Dual use research of concern
☒	☐	Plants

Methods

n/a	Involved in the study	
☒	☐	ChIP-seq
☒	☐	Flow cytometry
☒	☐	MRI-based neuroimaging

Antibodies

Antibodies used	anti-E-Cadherin (Cell Signaling Technology Cat no: 3195T), Goat anti-Rabbit (H+L) Highly Cross-Adsorbed Secondary Antibody, Alexa Fluor Plus 488 (Invitrogen, A32731TR)
Validation	E-Cadherin (24E10) Rabbit mAb detects endogenous levels of total E-cadherin protein. The antibody does not cross-react with related family members, such as N-cadherin. (Source: manufacturer's website). The goat anti-rabbit IgG whole antibodies have been pre cross-adsorbed against bovine IgG, goat IgG, mouse IgG, rat IgG, and human IgG, to minimize cross-reactivity. (Source:manufacturer's website)

Eukaryotic cell lines

Policy information about cell lines and Sex and Gender in Research

Cell line source(s)	MCF-7 (HTB-22, ATCC), A549 (CCL-185, ATCC), MDA-MB-231 (HTB-26, ATCC), MDCK (CCL-34, ATCC), Calu-3 (HTB-55, ATCC), U-2 OS (HTB-96, ATCC), T84 (CCL-248, ATCC), HaCaT (T0020001, AddexBio), HCT116 (CCL-247, ATCC), HepG2 (HB-8065, ATCC), K-562 (CCL-243), HT-29 (HTB-38), HBEC-5i (CRL-3245), hCMEC/D3 (SCC066, Sigma Aldrich)
Authentication	None of the cell lines were authenticated
Mycoplasma contamination	Cell lines were certified as mycoplasma negative from ATCC. Mycoplasma contamination was not tested internally.
Commonly misidentified lines (See ICLAC register)	None
